# Affective organizational commitment among nursing home employees: A longitudinal study on the influence of a health‐promoting work environment

**DOI:** 10.1002/nop2.338

**Published:** 2019-07-29

**Authors:** Karoline Grødal, Siw Tone Innstrand, Gørill Haugan, Beate André

**Affiliations:** ^1^ Department of Public Health and Nursing, Faculty of Medicine and Health Science NTNU – Norwegian University of Science and Technology Trondheim Norway; ^2^ NTNU Center for Health Promotion Research Trondheim Norway

**Keywords:** affective organizational commitment, eldercare, emotional demands, health care, job demands, job resources, nursing, nursing homes, work‐related sense of coherence

## Abstract

**Aim:**

To investigate whether affective organizational commitment (AOC) among nursing home employees is enhanced by a health‐promoting work environment, conceptualized as high levels of job resources, work‐related sense of coherence (work‐SOC) and low levels of job demands.

**Design:**

This study used a longitudinal design. Survey data were collected with a 1‐year interval between 2015/2016–2016/2017 among nursing home employees in Norway.

**Methods:**

Structural equation modelling was used to analyse the longitudinal data (*N* = 166) and cross‐sectional data from the first time point (*N* = 558).

**Results:**

The results supported that work‐SOC was strongly and positively related to AOC. Job resources and job demands were positively and negatively related, respectively, to work‐SOC but were not related to future AOC. The indirect effects of autonomy and supervisor support on AOC, via work‐SOC, were significant. The indirect effects regarding social community at work, emotional demands and role conflict were unclear.

## 1 INTRODUCTION

Eldercare services are facing challenges due to a steadily ageing world population and increasing number of persons in need of long‐term care (World Health Organization, 2014). In light of concerns regarding high nursing turnover (Hayes et al., 2012), the issue seems even more critical. Recruiting and retaining qualified and productive eldercare personnel will be crucial in the years to come. For nursing home leaders, one approach may be to promote employees' commitment to their organization. In general, commitment refers to: “a force that binds an individual to a target and to a course of action of relevance to that target” (Meyer, Becker, & van Dick, 2006, p. 666). Research has shown that organizational commitment is related to outcomes such as lower turnover intention (Graf, Cignacco, Zimmermann, & Zúñiga, 2016; Karsh, Booske, & Sainfort, 2005; Mathieu & Zajac, 1990; Meyer, Stanley, Herscovitch, & Topolnytsky, 2002), actual turnover (Mathieu & Zajac, 1990; Meyer et al., 2002) and absenteeism (Graf et al., 2016). Furthermore, higher job performance (Mathieu & Zajac, 1990) and quality of care among nursing home employees (Graf et al., 2016) have been reported.

The present study focuses on affective organizational commitment (AOC), which reflects that employees are emotionally attached to, can identify with and are involved in a particular organization (Meyer & Allen, 1991). To be able to focus interventions on strengthening employees' AOC, it is essential to know how AOC develops. For this purpose, it is most relevant to address work‐related precursors, which seem to have more influence on AOC than demographic and personal characteristics (Meyer et al., 2002). Perceived organizational support, organizational justice and transformational leadership are among the factors that have been shown to be strongly associated with AOC (Meyer et al., 2002).

Intervening on work characteristics is also relevant to influence workers' health (Bakker & Demerouti, 2014; Lesener, Gusy, & Wolter, 2019), suggesting that focusing on a healthy work environment might serve multiple purposes. The aim of the current longitudinal study was to investigate whether a health‐promoting work environment enhances AOC among nursing home employees in Norway. The job demands‐resources (JD‐R) model will serve as a theoretical framework for this and specific job demands, and resources are therefore measured to indicate characteristics of the work environment that are related to health impairment and enhancement, respectively. In addition, this study extends the literature by investigating the role of the more recent concept of work‐related sense of coherence (work‐SOC) in this context. Work‐SOC refers to the health‐promoting quality of an individual's work situation, reflected through the dimensions of how one perceives the environment as comprehensible, manageable and meaningful (Vogt, Jenny, & Bauer, 2013). To our knowledge, this is the first study investigating work‐SOC in relation to AOC.

### 1.1 Background

According to the JD‐R model (Bakker & Demerouti, 2007, 2017; Demerouti, Bakker, Nachreiner, & Schaufeli, 2001), all work characteristics can be labelled as either job demands or job resources. Those aspects of the job that require sustained physical or mental effort and are therefore associated with certain costs are categorized as job demands. Conversely, job resources contribute to achieving work goals, reducing job demands and their associated costs, or stimulating personal growth, learning and development. A central proposition of the JD‐R model is that job demands and job resources elicit health‐impairing and motivational processes, respectively. Through the motivational process, job resources contribute to work engagement and subsequent positive outcomes, whereas the health impairment process leads to negative outcomes via burnout. Meta‐analyses have supported these assumptions (Crawford, LePine, & Rich, 2010; Lesener et al., 2019), and previous studies have indicated that AOC is influenced by both of the above processes. Different job demands and job resources have been found to be directly associated with AOC (Mathieu & Zajac, 1990). In addition, Llorens, Bakker, Schaufeli, and Salanova (2006) found that burnout and work engagement partially mediated the relationship between work characteristics and organizational commitment.

The job demands investigated in this study are role conflict and emotional demands. Role conflict comprises inconsistent or conflicting information concerning demands at work (Nixon, Mazzola, Bauer, Krueger, & Spector, 2011), while emotional demands have to do with emotionally charged interaction with, for example, patients, or requirements to comply with certain rules about how to express feelings at work (Heuven, Bakker, Schaufeli, & Huisman, 2006). Previous studies among healthcare workers have shown that role conflict and emotional demands are related to outcomes such as frequent short‐term sick leave (Stapelfeldt et al., 2013) and burnout (Borritz et al., 2005; Piko, 2006). This study also investigates three job resources, namely, autonomy, supervisor support and social community at work. Autonomy has been widely studied and recognized as an important variable for organizational well‐being and performance (Nielsen et al., 2017). While supervisor support is about the degree to which the employee experiences instrumental or emotional support from his/her supervisor, social community has to do with a more “general perception of community spirit and reciprocity between colleagues sharing the same workplace” (Francioli et al., 2018, p. 891).

The theory of salutogenesis aims to explain the sources of health. The core of this theory is the concept of SOC, which refers to a global orientation to view one's internal and external environments as comprehensible, manageable and meaningful (Antonovsky, 1979, 1987). According to salutogenesis, SOC is essential for having the ability and capacity to understand and find meaning in one's situation to obtain better health. SOC enables people to identify and reflect on their internal and external resources and use them to cope with stressors and find solutions (Eriksson & Lindström, 2006). Studies have shown that SOC is related to work‐related outcomes such as occupational well‐being (Feldt, Kinnunen, & Mauno, 2000), work engagement (Vogt, Hakanen, Jenny, & Bauer, 2016), fewer stress symptoms (Albertsen, Nielsen, & Borg, 2001) and lower absence rates (Kivimäki et al., 1997). Strümpfer and Mlonzi (2001) reported a weak, although significant, correlation between SOC and organizational commitment.

More recently, a work‐related SOC concept (work‐SOC) was proposed, where the three dimensions were assumed to reflect aspects of an individual's current work situation (Vogt et al., 2013). Comprehensibility reflects a work situation perceived as structured, consistent and clear; manageability is the perception of having adequate resources available to cope with job demands; and meaningfulness involves seeing work as worthy of commitment and involvement (Vogt et al., 2013). In addition to the work environment, individual characteristics and previous experiences are thought to influence the perception of these dimensions, which means that work‐SOC is theoretically more sensitive to change than the global SOC. In addition, work‐SOC seems to be a better predictor for work engagement than the global SOC (van der Westhuizen, 2018) and it is assumed that this is the case for other work‐related outcomes as well.

It has been argued that work‐SOC and the associated salutogenic theory might contribute to the JD‐R model with a more explicit focus on health; this focus would be not only on health impairment but also on the path from job resources to positive outcomes (Brauchli, Jenny, Füllemann, & Bauer, 2015; Jenny, Bauer, Vinje, Vogt, & Torp, 2017). It is assumed that job demands and job resources have negative and positive influences, respectively, on work‐SOC, which subsequently affect health‐ and work‐related outcomes. Vogt et al. (2013) found that work‐SOC acted as a partial mediator of the cross‐sectional relationships between job demands and exhaustion and between job resources and work engagement. Based on this, we assume that work‐SOC will also have a positive influence on AOC and that work‐SOC is a mediator between work characteristics (job demands and resources) and AOC.

The aim of the current study was to investigate whether a health‐promoting work environment enhances AOC among nursing home employees in Norway. More specifically, we investigate how AOC is influenced by job demands (role conflict and emotional demands), job resources (autonomy, supervisor support and social community at work) and work‐SOC.

**Figure 1:**
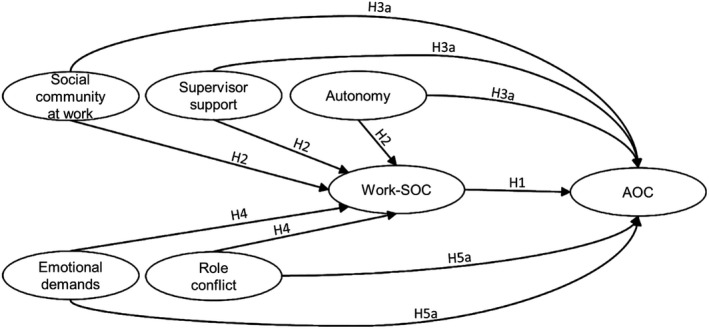
Hypothesized relationships between the study variables

Based on the background that has been presented, the relationships between the study variables are hypothesized as follows (see also Figure 1):

H1: Work‐SOC is positively related to AOC.

H2: Job resources (autonomy, supervisor support and social community at work) are positively related to work‐SOC.

H3: Job resources (autonomy, supervisor support and social community at work) are positively related to AOC (a) directly and (b) indirectly through work‐SOC.

H4: Job demands (emotional demands and role conflict) are negatively related to work‐SOC.

H5: Job demands (emotional demands and role conflict) are negatively related to AOC (a) directly and (b) indirectly through work‐SOC.

## 2 METHODS

### 2.1 Design

A longitudinal design was applied in the current study.

### 2.2 Data collection procedure

The data for the present study were collected in two waves, with a 1‐year interval, among employees from 43 nursing homes in two Norwegian municipalities. In regard to potential seasonal variations in the nursing homes, data collection was set to the same period each year. The same data collection procedure was followed at both time points. Contact persons in each nursing home distributed e‐mails with information about the study and invitations to participate in an online survey. The survey was completed by 558 employees at the first time point (T1) and 515 at the second time point (T2). Responses at T1 and T2 were linked by personal codes created by the respondents, leaving a sample of 166 employees who completed the survey at both time points.

Based on the numbers of invitations sent (2,835 and 3,221 at T1 and T2, respectively), the response rates were estimated to be 20% and 16%, respectively. However, the response rates were probably higher in reality. Some contact persons noted that past employees might have received invitations because their mailing lists were not recently updated and that some employees might have received multiple invitations because of multiple employments. In addition, the nursing homes seemed to have different practices regarding e‐mail communication, meaning that we could not be certain that all invitations were read.

### 2.3 Participants

In the current study, the cross‐sectional sample with answers from T1 (*N* = 558) was applied, in addition to the longitudinal sample (*N* = 166). In the following, characteristics are presented for the two samples (cross‐sectional/longitudinal). A total of 90%/92% were women, and the age ranges were 17–72/20–66 years (mean = 42.1, *SD*: 13.1/mean = 44.9, *SD*: 12.1). A large female majority is representative of the population according to the statistics on health and social services in Norway (Statistics Norway, 2019). Professional groups were distributed between nurses (40%/45%), assistant nurses (38%/38%), other health‐ and social‐related personnel (19%/13%) and staff and support functions (2%/4%). Employees had a mean of 29.4/30.9 (*SD*: 9.6/8.2) work hours per week and tenure of 7.6/9.0 years (*SD*: 7.2/7.3) at their current workplace and 17%/19% of employees had leadership responsibilities.

### 2.4 Instruments

Job demands were measured by two five‐point scales from the Copenhagen Psychosocial Questionnaire (COPSOQ; Pejtersen, Kristensen, Borg, & Bjorner, 2010), which have shown good properties for application within the JD‐R framework (Berthelsen, Hakanen, & Westerlund, 2018). *Emotional demands* were measured by four items (e.g., “Does your work put you in emotionally disturbing situations?”). *Role conflict* was measured by four items (e.g., “Are contradictory demands placed on you at work?”).

Job resources were measured by three five‐point scales. Two were from the COPSOQ (Pejtersen et al., 2010). *Social community at work* was measured by three items (e.g., “Is there a good atmosphere between you and your colleagues?”). *Supervisor support* was measured by three items (e.g., “How often do you get help and support from your nearest superior?”). The third job resource, *autonomy*, was measured by a five‐point scale with four items (e.g., “There is scope for me to take own initiatives in my work”) by Sverke and Sjöberg (1994), based on Hackman and Oldham (1975) and Walsh, Taber, and Beehr (1980).

*Work‐SOC* was measured using a scale where employees were asked to rate how they perceived their current job and work situation in general on a seven‐point scale between bipolar adjective pairs (Bauer, Vogt, Inauen, & Jenny, 2015; Vogt et al., 2013). The scale was treated according to a previous validation of the Norwegian translation of the scale (Grødal et al., 2018) with three items representing comprehensibility (e.g., “Structured – Unstructured”), two items representing manageability (e.g., “Easy to influence – Impossible to influence”) and three items representing meaningfulness (e.g., “Meaningless – Meaningful”).

*AOC* was measured by a scale from the COPSOQ (Pejtersen et al., 2010). Four items (e.g., “Do you enjoy telling others about your place of work?”) were rated on a five‐point scale (from “To a very large extent” to “To a very small extent”).

### 2.5 Analysis

All statistical analyses in the current study were conducted using Stata version 15.1 (StataCorp, 2017). Hypotheses 1–5 were tested by means of a structural equation modelling (*SEM*) approach with maximum likelihood estimation. Missing values were deleted listwise in all analyses. In addition to chi‐square (*χ* ^2^), the following criteria were used to evaluate goodness of fit: root mean squared error of approximation (RMSEA) <.06, standardized root mean squared residual (SRMR) <.08 and comparative fit index (CFI) and Tucker–Lewis index (TLI) close to .95. These values were not regarded as exact cut‐off values because these criteria may be overly strict under conditions of non‐robust data and small samples (Hu & Bentler, 1999), as was apparent, especially, in the longitudinal sample in the current study. The direct, indirect and total effects were estimated for six models: one cross‐sectional model and five separate longitudinal models for each job demand and job resource. The rationale behind this approach was that the longitudinal sample size was too small to test the full model and model complexity (i.e., number of estimated parameters) therefore had to be minimized to obtain adequate power. The full model was tested with cross‐sectional data to investigate the hypotheses taking all job resources and job demands into account.

Prior to investigating the hypotheses, the measurement models were specified and tested according to our data. The cross‐sectional model (M1), using data from T1, included AOC and the specific job demands and job resources indicated by their respective scale items, as well as work‐SOC indicated by the mean scores of the subscales of comprehensibility, manageability and meaningfulness as suggested by Grødal et al. (2018). Each longitudinal model (M2–M6) included one of the specific job demands/resources indicated by its respective scale items at T1, AOC measured at both T1 and T2 with respective scale items as indicators and work‐SOC change indicated by the standardized residual scores of comprehensibility, manageability and meaningfulness. The standardized residuals were obtained by regressing the mean scores at T2 on their corresponding scores at T1. The residual scores indicate whether employees' work‐SOC has changed more (positive values) or less (negative values) than expected based on their score at T1 (Cronbach & Furby, 1970). This approach has been used in previous studies with similar variables and designs (Schaufeli, Bakker, & van Rhenen, 2009; Tims, Bakker, & Derks, 2013).

Some modifications were made to ensure the quality of the measurement models. Due to convergent validity problems with the latent constructs of role conflict (average variance extracted, AVE = 0.492) and emotional demands (AVE = 0.496), the models were adjusted by removing two items: the role conflict item, “Do you do things at work, which are accepted by some people but not by others” and the emotional demands item, “Do you have to relate to other people's personal problems as part of your work?” These were the indicators with the poorest factor loadings on their respective latent variables, and modification indices suggested cross‐loadings on some of the other variables in the model. Theoretically, the removed role conflict item was interpreted to differ from the other three because it says something about how other people judge employees' actions at work, while the other items ask more directly about how the employee himself or herself perceives the conflicting demands that are placed on them. Regarding the removed emotional demands item, this item seemed to differ in that it asked about specific situations, while the remaining items concerned whether work was emotionally demanding in a more general sense. Lastly, the measurement errors of comprehensibility and manageability were allowed to covary based on strong suggestions from modification indices in all models, also supported by the results from a previous validation indicating poor discriminant validity between these dimensions of work‐SOC (Grødal et al., 2018).

### 2.6 Ethics

The Norwegian Centre for Research Data, Data Protection Services, was notified of the project. Prior to this, an application was sent to the Regional Committee for Medical and Health Research Ethics, who declared that approval for the current project was not required according to the Norwegian Health Research Act. Participation in the study was voluntary, and the employees gave their consent by completing the survey. Data were kept confidential and will be anonymized at the end of the project.

## 3 RESULTS

To check for potential attrition bias, dropouts (considered those who answered the survey at T1 but not T2) and those who answered the survey at both time points were compared based on the relevant variables. Dropouts were significantly younger (*t*(525) = −3.46, *p* < .001; *d* = −0.32) and had fewer contracted work hours per week (*t*(510) = −2.35, *p* < .05; *d* = −0.22). Dropouts also scored lower on work‐SOC (*t*(525) = −3.70, *p* < .001; *d* = −0.35), autonomy (*t*(549) = −2.62, *p* < .01; *d* = −0.24) and social community at work (*t*(553) = −2.51, *p* < .01;* d* = −0.18). No significant differences were found regarding AOC, supervisor support, emotional demands and role conflict.

Tables 1 and 2 show the means, standard deviations, internal consistencies and bivariate correlations (Pearson's *r*) among the study variables for both samples. All bivariate correlations relevant to the study hypotheses were significant and in the expected directions. The goodness of fit of the study models is presented in Table 3. Overall, the models did not show optimal, though acceptable, fit to the data because there were some deviations from the aforementioned cut‐offs.

**Table 1:** Means (*M*), standard deviations (*SD*), internal consistencies and correlations between the study variables in the cross‐sectional sample (*N* = 558)

	*M*	*SD*	1	2	3	4	5	6	7
1. AOC T1	3.73	0.88	(.83)						
2. Work‐SOC T1	5.06	1.14	.58***	(.87)					
3. Autonomy T1	3.45	0.75	.50***	.47***	(.83)				
4. Supervisor support T1	3.40	0.95	.56***	.40***	.42***	(.85)			
5. Social community at work T1	4.23	0.66	.52***	.43***	.34***	.43***	(.80)		
6. Emotional demands T1	3.31	0.68	−.22***	−.17***	−.20***	−.12**	−.09*	(.76)	
7. Role conflict T1	2.56	0.79	−.44***	−.33***	−.30***	−.34***	−.31***	.45***	(.78)

Note

Internal consistencies are Cronbach's alphas (*α*) in diagonals. Correlations are Pearson's *r*.

Abbreviations: AOC, affective organizational commitment; work‐SOC, work‐related sense of coherence.

* *p* < .05.

** *p* < .01.

*** *p* < .001.

**Table 2:** Means (*M*), standard deviations (*SD*), internal consistencies and correlations between the study variables in the longitudinal sample (*N* = 166)

	*M*	*SD*	1	2	3	4	5	6	7	8	9	10
1. AOC T1	3.84	0.88	(.86)									
2. Work‐SOC T1	5.34	1.13	.70***	(.87)								
3. Autonomy T1	3.57	0.71	.51***	.41***	(.82)							
4. Supervisor support T1	3.51	0.94	.68***	.49***	.52***	(.85)						
5. Social community at work T1	4.33	0.57	.58***	.47***	.40***	.50***	(.78)					
6. Emotional demands T1	3.31	0.72	−.27***	−.29**	−.24**	−.15	−.23**	(.81)				
7. Role conflict T1	2.46	0.73	−.54***	−.37***	−.33***	−.36***	−.37***	.51***	(.76)			
8. AOC T2	3.82	0.89	.79***	.60***	.52***	.56***	.53***	−.23**	−.49***	(.86)		
9. Work‐SOC T2	5.41	1.10	.56***	.59***	.41***	.42***	.33***	−.28***	−.42***	.68***	(.87)	
10. Work‐SOC change	0.00	1.00	.20**	−.00	.18*	.17*	.06	−.19*	−.23**	.41***	.81***	(.81)

Note

Internal consistencies are Cronbach's alphas (*α*) in diagonals. Correlations are Pearson's *r*. Work‐SOC change represents standardized residual scores obtained by regressing work‐SOC T2 on work‐SOC T1.

Abbreviations: AOC, affective organizational commitment; work‐SOC, work‐related sense of coherence.

* *p* < .05.

** *p* < .01.

*** *p* < .001.

**Table 3:** Goodness of fit of structural equation models

	*N*	*χ* ^2^ (*df*)	*χ* ^2^/*df*	RMSEA	SRMR	CFI	TLI
M1 cross‐sectional	483	482.513 (208)***	2.320	.052	.054	.949	.938
M2 longitudinal, autonomy	151	159.540 (80)***	1.994	.081	.068	.943	.925
M3 longitudinal, supervisor support	150	113.752 (67)***	1.728	.068	.053	.966	.954
M4 longitudinal, social community at work	148	115.266 (67)***	1.720	.070	.070	.962	.948
M5 longitudinal, emotional demands	152	128.493 (67)***	1.918	.078	.105	.952	.935
M6 longitudinal, role conflict	151	113.763 (67)***	1.698	.068	.057	.964	.951

Abbreviations: CFI, comparative fit index; *df*, degrees of freedom; RMSEA, root mean squared error of approximation; SRMR, standard root mean squared residual; TLI, Tucker–Lewis index; *χ* ^2^, chi‐squared.

*** *p* < .001.

Table 4 displays the standardized estimates of the direct and indirect effects obtained from the cross‐sectional and longitudinal analyses. The cross‐sectional and longitudinal analyses were consistent in finding positive relationships between work‐SOC and AOC (H1) and between job resources and work‐SOC (H2), whereas the other hypotheses received partial support. None of the job demands or job resources were directly associated with T2 AOC when T1 AOC and work‐SOC change were controlled for (H3a and H5a). However, the cross‐sectional analysis showed that supervisor support and social community at work were significantly positively related to AOC, while role conflict was negatively related to AOC (H3a and H5a). Emotional demands and role conflict were significantly negatively related to work‐SOC in the longitudinal analyses, while this finding only pertained to role conflict in the cross‐sectional analysis where the other work characteristics were controlled for (H4). The cross‐sectional analyses showed that all three job resources, but none of the job demands, had indirect effects on AOC through work‐SOC (H3b and H5b). The longitudinal analyses supported the indirect effects of autonomy, supervisor support, emotional demands and role conflict but not social community at work (H3a and H3b).

**Table 4:** Standardized estimates of direct and indirect effects

Path		Model					
Endogenous variable	Exogenous variable	1	2	3	4	5	6
Direct effects							
Work‐SOC ←	Autonomy	.441***	.476**				
	Supervisor support	.158*		.436**			
	Social community at work	.258***			.420*		
	Emotional demands	−.005				−.345*	
	Role conflict	−.172*					−.503**
AOC ←	AOC		.701***	.828***	.717***	.811***	.754***
	Work‐SOC	.585***	.532*	.559*	.516*	.550**	.581*
	Autonomy	.013	−.049				
	Supervisor support	.152*		−.237			
	Social community at work	.151*			−.049		
	Emotional demands	.060				.150	
	Role conflict	−.143*					.159
Indirect effects							
AOC ←	Autonomy	.258*	.253*				
	Supervisor support	.099*		.244*			
	Social community at work	.151*			.217		
	Emotional demands	−.003				−.190*	
	Role conflict	−.101					−.292*

Note

Model 1 analysed with cross‐sectional data from T1. Models 2–6 analysed with longitudinal data with job demands and job resources from T1, work‐SOC change from T1–T2 and AOC from T1–T2. Indirect effects via work‐SOC.

Abbreviations: AOC, affective organizational change; work‐SOC, work‐related sense of coherence.

* *p* < .05.

** *p* < .01.

*** *p* < .001.

## 4 DISCUSSION

The aim of the current study was to investigate whether AOC among nursing home employees in Norway is enhanced by a health‐promoting work environment, conceptualized by high levels of job resources and work‐SOC and low levels of job demands. The main finding was that work‐SOC was consistently found to be strongly positively related to AOC. Additionally, the results fully supported that job resources were positively related to work‐SOC and that role conflict was negatively related to work‐SOC. However, none of the job demands or job resources were significantly related to AOC at T2 when AOC at T1 and work‐SOC change were controlled for. Only cross‐sectional relationships regarding supervisor support, social community at work and role conflict were detected, while autonomy and emotional demands were not directly related to AOC at all. The indirect effects of work‐SOC on AOC were consistent regarding autonomy and supervisor support but more unclear regarding the other variables. The main focus of the discussion will be on the strong support for work‐SOC as a predictor and precursor of AOC, in addition to the case of emotional demands, which yielded some unexpected results.

### 4.1 Main findings and theoretical implications

The results of the present study consistently and strongly suggest that work‐SOC is a better predictor for both current and future AOC than single job resources and job demands. The longitudinal analyses showed that work‐SOC was actually the only significant predictor of AOC when AOC at T1 was controlled for. This result indicates that work‐SOC is important in the development of AOC and is a relevant indicator to consider for nursing home leaders. However, all hypotheses in the study model were not entirely confirmed, which means that the theoretical explanations for the relationship between work‐SOC and AOC need to be discussed.

Based on the JD‐R model and salutogenic theory, it was hypothesized that work characteristics would be related to work‐SOC, which further affected AOC. A positive influence was assumed through a motivational or salutogenic pathway where job resources affected AOC directly and indirectly through work‐SOC. A corresponding negative influence was assumed, with job demands as the starting point. To a certain degree, these propositions were confirmed. However, none of the investigated work characteristics seemed to be directly related to AOC at a later stage. One explanation can be that the variance of the single work characteristics that are relevant to AOC is shared by the more comprehensive factor of work‐SOC, and thereby, such characteristics lose their predictive power.

Additionally, work‐SOC, in the framework of the JD‐R model, could more accurately be labelled as a personal resource than as its own category, as was first assumed (Jenny et al., 2017). Personal resources have been defined as the degree to which people believe to have control over their environment and are thought to have reciprocal positive effects on job resources (Bakker & Demerouti, 2017). In addition, personal resources are thought to work in the same manner as job resources in creating positive effects and buffering the negative effects of job demands. These propositions were not tested in this study but seem plausible and cannot be ruled out. The results of a previous study found that the relationship between emotional demands and work engagement was weak or not significant among employees with a high degree of personal resources (Xanthopoulou, Bakker, & Fischbach, 2013), and the sample in the current study indeed had a high mean score on work‐SOC.

These considerations also start the discussion regarding emotional demands, which, contrary to the hypotheses, did not seem to have any effect on either work‐SOC or AOC. The exception was a significant negative relationship with work‐SOC in the longitudinal analysis, which did not account for the other job demands or job resources. The lack of relationship between emotional demands and AOC replicates a previous finding by Clausen and Borg (2010), who also conducted their study among employees in eldercare. They suggested that emotionally demanding work may also be characterized by factors contributing positively to AOC, such as meaning in work or intrinsic job rewards (Clausen & Borg, 2010), which might be a plausible explanation considering our results, which indicated that work‐SOC (incorporating meaningfulness) had a weaker negative relationship with emotional demands than role conflict. In fact, the cross‐sectional *SEM* did not show any relationship between emotional demands and work‐SOC, which could imply that different effects had balanced each other out.

There are several plausible explanations for the lack of relationships between emotional demands and AOC. First, one explanation might be that employees in the healthcare sector expect to meet emotional demands and that the negative effects are less apparent than they would be if the demands were unexpected. Second, since emotional demands are inherent in healthcare work, one explanation might be that resources to counteract their negative effects are more likely to be in place. For example, our results show that employees score particularly high on social community at work. We also see that the bivariate correlations between emotional demands and supervisor support are not significant among the longitudinal sample, while the JD‐R model suggests that the relationships between job demands and job resources should be negative (Bakker & Demerouti, 2007). Third, another explanation might be that nursing home employees perceive emotional demands not only as hindrances but also as challenges (Zapf, 2002). Challenging demands do not only induce strain but also provide opportunities for performance and accomplishment (Webster, Beehr, & Love, 2011), which in turn might be positively related to AOC. The differentiation between hindrance and challenging job demands is yet to be incorporated into the JD‐R model because of a lack of knowledge (Bakker & Demerouti, 2017), and the current study highlights that these mechanisms should be investigated more in future research.

### 4.2 Limitations

The use of longitudinal data, validated instruments and advanced statistics was among the strengths of this study. However, there were also limitations that must be taken into account. First, *SEM* is a method that requires relatively large samples (Kline, 2011). Optimally, the full model should have been tested with longitudinal data, but relatively few respondents answered the survey at both time points. We therefore chose to test the full model with cross‐sectional data, while longitudinal data were used by testing separate models for each job demand and job resource. A limitation of this approach is that the other job demands and job resources were not controlled for in the longitudinal analyses. The sample size and corresponding small group sizes of the 43 nursing homes were also a reason for disregarding a multilevel analysis approach, which was relevant given the nested data structure.

Second, the response rate was low, which is a common challenge in studies among health personnel (Fida, Laschinger, & Leiter, 2018; van der Heijden, Demerouti, Bakker, & Hasselhorn, 2008; Mark & Smith, 2012), possibly affecting the generalizability of the findings. Attrition bias might have affected the results to a certain degree. Dropouts scored lower than respondents who participated at both time points, concerning the variables of work‐SOC, autonomy and social community at work, potentially due to a healthy worker effect. Additionally, dropouts were younger and had fewer contracted work hours, meaning that turnover could be a likely explanation (Hayes et al., 2012). However, we had no data to test this assumption. Third, all data were based on self‐reports, meaning that common method bias might be present (Podsakoff, MacKenzie, Lee, & Podsakoff, 2003). On the other hand, time lags might have reduced this effect (Doty & Glick, 1998). Fourth, even the longitudinal design does not guarantee that causal inferences can be made from this study (Spector, 2019). However, the combined cross‐sectional and longitudinal approaches contribute to extend the understanding of potential mechanisms explaining the relationships between the study variables.

## 5 CONCLUSION

Taken together, the results of this study provide support for the assumption that work‐SOC enhances AOC among nursing home employees. However, the influence of specific job demands and resources in this context seems more unclear. Suggestions for future research are to clarify the role of emotional demands and how they might potentially contribute to both positive and negative outcomes for employees and to look more closely at the mechanisms surrounding the development of work‐SOC and what explains its association with AOC.

## CONFLICT OF INTEREST

We have no conflict of interest to declare.

## AUTHOR CONTRIBUTIONS

KG, STI, BA and GH: Substantial contributions to conception and design, or acquisition of data, or analysis and interpretation of data; drafting the manuscript or revising it critically for important intellectual content; approval of the version to be published; and agreed to be accountable for all aspects of the work in ensuring that questions related to the accuracy or integrity of any part of the work are appropriately investigated and resolved. Each author should have participated sufficiently in the work to take public responsibility for appropriate portions of the content.
